# Potenziale und Herausforderungen von sozialen Robotern für Beziehungen älterer Menschen: eine Bestandsaufnahme mittels „rapid review“

**DOI:** 10.1007/s00391-021-01932-5

**Published:** 2021-07-06

**Authors:** Jan C. Zöllick, Susanna Rössle, Lina Kluy, Adelheid Kuhlmey, Stefan Blüher

**Affiliations:** 1 Institut für Medizinische Soziologie und Rehabilitationswissenschaft, Charité – Universitätsmedizin Berlin, corporate member of Freie Universität Berlin and Humboldt-Universität zu Berlin, Charitéplatz 1, 10117 Berlin, Deutschland; 2 Institut für Philosophie, Freie Universität Berlin, Habelschwerdter Allee 30, 14195 Berlin, Deutschland; 3 Karlsruher Institut für Technologie, Institut für Arbeitswissenschaft und Betriebsorganisation, Engler-Bunte-Ring 4, 76131 Karlsruhe, Deutschland

**Keywords:** Pflege und Roboter, Gerontotechnologie, Hochaltrige, Soziale Netzwerke, Einsamkeit, Care and robots, Gerontotechnology, Old age, Social networks, Loneliness

## Abstract

**Hintergrund:**

Soziale Beziehungen sind bedeutsame Ressourcen für psychisches Wohlbefinden und physische Gesundheit. Im höheren Lebensalter treffen zunehmende Vulnerabilität und Funktionsverluste häufig auf reduzierte soziale Netzwerke. Mangelnde soziale Kontakte und fehlende Netzwerke bergen dabei psychische und physische Risiken für die Betroffenen, die durch den Einsatz sozialer Roboter möglicherweise abgemildert werden können.

**Fragestellung:**

Welche Potenziale und Herausforderungen ergeben sich für ältere Menschen aus ihrer Interaktion mit sozialen Robotern?

**Material und Methoden:**

Die Forschungsfrage wird mittels eines „rapid review“ beantwortet. Eine systematische Literatursuche ergab 433 unikale Treffer, aus denen *n* = 11 Artikel in die Analysen eingingen.

**Ergebnisse:**

Potenziale sozialer Roboter bestehen in der Reduktion von Einsamkeit, Stärkung der (zwischenmenschlichen) Kommunikation und Stimmungsaufhellung bei gleichzeitiger Stressreduktion. Herausforderungen bestehen in der sozialen Einbettung der Roboter. Diese sei durch Aspekte wie Wohltätigkeit, Autonomie und Privatheit als Grundsätze zu gestalten, an denen sich Design und Einsatz von sozialen Robotern orientieren können, um einem Verlust von sozialen Beziehungen vorzubeugen.

**Diskussion:**

Die Ergebnisse zeigen einen Korridor auf, der die potenzialausschöpfende Anwendung sozialer Roboter für ältere Menschen ermöglicht. Im Vordergrund steht die Analyse der Herausforderungen für den Einzelfall, da soziale Beziehungen älterer Menschen positiv sowie negativ beeinflusst werden können. Dabei orientieren sich die eingeschlossenen Artikel größtenteils am Setting Pflege. Forschung zum Einsatz sozialer Roboter bei nicht oder wenig funktionseingeschränkten Personen sollte die bestehende Literatur ergänzen.

**Zusatzmaterial online:**

Zusätzliche Informationen sind in der Online-Version dieses Artikels (10.1007/s00391-021-01932-5) enthalten.

## Abstract (en)

**Background:**

Social relationships are essential resources for psychological well-being and physical health. In old age, increased vulnerability and loss of functions are accompanied by diminishing social networks reinforcing a vicious circle. This decrease of social cohesion presents risks for health and well-being that might be cushioned by introducing social robots.

**Objective:**

What potentials and challenges for older people result from their interaction with social robots?

**Material und methods:**

The objectives are approached via a rapid review. The systematic search resulted in 433 unique articles. Of these 11 articles were included in the analysis.

**Results:**

Social robots have the potential to reduce loneliness, reinforce (interpersonal) communication, enhance the mood while reducing stress. Challenges lie in the social embedding of the interaction with robots. Authors suggest principles such as charity, autonomy, and privacy as helpful guidelines for the design and use of social robots to prevent a loss of social relationships.

**Conclusion:**

The results present corridors for the viable use of social robots for older adults offering to exploit existing potentials. At the core lies a view on the individual case with its unique circumstances and predispositions, because social robots bear positive and negative outcomes regarding social relationships. The identified studies focused on deficient settings (e.g., particular disorders within care facilities). Thus, research on the use of social robots by healthy adults should add to the existing literature.

## Hintergrund und Fragestellung

Das höhere Lebensalter ist vielfach durch eine Verkleinerung sozialer Netzwerke gekennzeichnet [10]. Gleichzeitig stellen soziale Partizipation, Kommunikation sowie der Austausch von Unterstützungsleistungen bedeutsame Ressourcen für psychisches Wohlbefinden und physische Gesundheit dar. Der aktiven Gestaltung sozialer Beziehungen kommt daher in jeder Lebensphase, ganz besonders jedoch angesichts erhöhter Vulnerabilität im Alter, große Bedeutung zu. Bei zunehmender Technisierung und Digitalisierung von Lebensbereichen stellen sich auch Fragen nach Einsatzmöglichkeiten spezifischer technischer Anwendungen in Bezug auf Partizipation, Kommunikation und Beziehungsgestaltung. Ziel dieses Beitrags ist es, die in der Fachliteratur beschriebenen Potenziale und Herausforderungen sozialer Roboter im Hinblick auf Beziehungen älterer Menschen zu diskutieren und den diesbezüglichen Forschungsstand überblicksartig darzustellen.

Soziale Beziehungen sind eine entscheidende Ressource für die Lebenszufriedenheit, das Gesundheitshandeln und den Gesundheitsstatus im Leben, das durch die konstante Auseinandersetzung mit Abhängigkeiten zu anderen Menschen gekennzeichnet ist [17]. In einer Metaanalyse von 70 Studien wurde gezeigt, dass sich soziale Isolation und Einsamkeit negativ, soziale Vernetzung hingegen positiv auf das Wohlbefinden und die Lebenserwartung auswirken [10]. Damit sind soziale Beziehungen wesentliche Ressourcen, die Stress verringern und die Lebensqualität erhöhen [20]. Eine der wesentlichen protektiven Funktionen sozialer Beziehungen besteht in der Reduktion von Einsamkeit – dem subjektiven, negativen Gefühl des Nichtverbundenseins, das auch in tatsächlicher Interaktion mit anderen Menschen auftreten kann und somit nicht eindeutig an objektive Faktoren wie Kontakthäufigkeit gebunden ist [10]; jedoch erhöhen kleiner werdende Netzwerke die Gefahr von Isolation und Einsamkeit. Kleinere Netzwerke bedeuten nicht in allen Fällen, dass soziale Interaktion verringert wird; die Qualität und Intensität eines sozialen Kontakts können nicht mit der Häufigkeit sozialer Interaktion gleichgesetzt werden. Nichtsdestotrotz hängt gefühlte Einsamkeit oft mit wenig sozialer Interaktion zusammen [10]. Über die Lebensspanne verändern sich soziale Netzwerke, die durchschnittliche Größe privater Netzwerke nimmt dabei bereits ab einem Maximum im vierten Lebensjahrzehnt ab [23]. Um drohende Ressourcenverluste ggf. zu kompensieren, könnten sich vermehrt technische Möglichkeiten der sozialen Robotik anbieten.

Soziale Roboter im engeren Sinne sind definiert als Roboter, die mit ihrer Umwelt auf solch eine selbstständige Weise kommunizieren und interagieren, dass Menschen ein soziales Modell nutzen, um ihr Verhalten zu erklären [3]. Soziale Roboter sehen meist menschen- oder tierähnlich aus, sodass Anthropomorphismus – die Zuschreibung von Emotionen, Intentionen und anderen menschlichen Charakteristika – vereinfacht und gefördert wird [7], bis hin zur Annahme eines eigenen Willens („agency“) der Maschinen [16]. Im Wesentlichen können soziale Roboter in zwei Kategorien eingeteilt werden [5]. Die erste Kategorie sieht den Roboter als Begleiter („companion“), der Gesellschaft leistet, an Unterhaltungen teilnimmt und mit dem Menschen „auf Augenhöhe“ interagiert bzw. sogar *für ihn sorgt*. In diese Kategorie fallen beispielsweise Pepper, ein mittelgroßer humanoider Roboter, oder auch der für ältere, zu Hause lebende Menschen entwickelte SYMPARTNER-Roboter [15]. Roboter der zweiten Kategorie sind nicht als Assistenten konzipiert, sondern vielmehr als Fürsorgeobjekt, *für das der Mensch sorgt*. Dabei werden Verantwortung geübt und idealerweise Kompetenzen aufrechterhalten. Ein Beispiel dieser Kategorie ist Paro, dem Vorbild einer Robbe nachempfunden und speziell für den therapeutischen Einsatz bei älteren Menschen konzipiert [8]. Paro kann durch Sensoren und Reaktoren auf Geräusche und Berührungen mit tierähnlichen Bewegungen und Lauten reagieren.

Bereits Dautenhahn [5] betrachtete soziale Roboter im Zusammenhang mit sozialen Beziehungen und zeigte, dass Interaktionen mit Robotern nach ähnlichen „sozialen Gesetzen“ wie menschliche Interaktionen stattfinden können, beispielsweise wenn sie im Kontext des gemeinsamen Spiels gesetzt werden, das als Grundlage des Beziehungsaufbaus genutzt wurde. Ebenso zeigten Meyer und Fricke [15], dass die Integration eines Roboters in das Zuhause älterer Menschen eine Dynamisierung des Alltagslebens nach sich zog. In diesem Paradigma werden soziale Roboter vermehrt für den Einsatz bei älteren Menschen entwickelt und genutzt. Da diese Zielgruppe durch eine im Schnitt höhere soziale und gesundheitsbezogene Vulnerabilität charakterisierbar ist, ist insbesondere hier weitere Forschung zu den Bedingungen und Auswirkungen des Einsatzes nötig, besonders im Hinblick auf soziale und ethische Aspekte. Aus soziologischer Sicht ergibt sich folgende Forschungsfrage:* Welche Potenziale und Herausforderungen ergeben sich für ältere Menschen aus ihrer Interaktion mit sozialen Robotern?*

## Studiendesign und Untersuchungsmethoden

Die Forschungsfrage wird mittels eines „rapid review“ beantwortet, definiert als „rigorous and transparent form of knowledge synthesis that accelerates the process of conducting a traditional systematic review through streamlining or omitting a variety of methods to produce evidence […] in a resource-efficient manner“ [9]. Im Vergleich zu einem „systematic review“ wurden dabei das „quality appraisal“ und das „risk of bias assessment“ der eingeschlossenen Studien übersprungen. Diese beiden Schritte ergeben sich besonders im Falle quantitativer Metaanalysen, die im vorliegenden Fall jedoch nicht durchgeführt wurden. Ein gewisser Zeitdruck der Thematik soziale Roboter ergibt sich, „da sich digitale Technikgenerationen überaus schnell verändern sowie erst wenige nachhaltige Standards und Richtlinien etabliert sind. Hierbei droht die Gefahr, dass in der Gerontologie und Geriatrie Wesentliches verpasst wird, denn das Momentum ist genau jetzt aktiv und jetzt gestaltbar“ [22]. Dieser Beitrag zielt darauf ab, auf der Basis der theoretischen und empirischen Literatur einen Überblick über die diversen Potenziale und Herausforderungen einer Nutzung sozialer Roboter bei älteren Menschen zu geben und hier bestehende Forschungslücken zu identifizieren. Dies soll die Debatte um soziale Roboter reflektieren und strukturieren.

Im Frühjahr 2020 wurden die Datenbanken *Web of Science* und *PubMed* mithilfe der folgenden Suchstrategie durchsucht: [elder*] AND [„social robot*“ OR „care robot*“ OR „assistive robot*“]. Alle Artikel wurden in eine „endnote library“ zusammengeführt und, basierend auf Titeln, Abstracts und Volltexten, nach ihrer inhaltlichen Passung zur Forschungsfrage ein- oder ausgeschlossen. Methodische Einschlusskriterien waren empirische und theoretische Artikel mit Begutachtungsverfahren in deutscher und englischer Sprache. Thematisches Einschlusskriterium waren die möglichen Auswirkungen des Einsatzes sozialer Roboter auf Beziehungsaspekte älterer Menschen. Studien im Pflegekontext mit besonderen Einschränkungen wie Demenzerkrankungen betrachten dabei Potenziale und Herausforderungen in eher defizitorientieren Settings; diese werden idealerweise ergänzt durch Studien in der eigenen Häuslichkeit und ohne schwerwiegende Funktionseinschränkungen. Einschränkungen zu (Gesundheits‑)Outcomes wurden nicht gemacht. Reviews, Buchkapitel und Konferenzbeiträge wurden ausgeschlossen. Zusätzlich zur Suchstrategie wurden Literaturverzeichnisse besonders aussagekräftiger Studien gesichtet. Die Analyse der Studien folgte dem Rapid review mit thematischem Fokus, d. h., die Studien werden nicht dezidiert diskutiert, sondern thematisch geordnet und im Sinne der Wissenssynthese wiedergegeben [21].

## Ergebnisse

**Figure 1:**
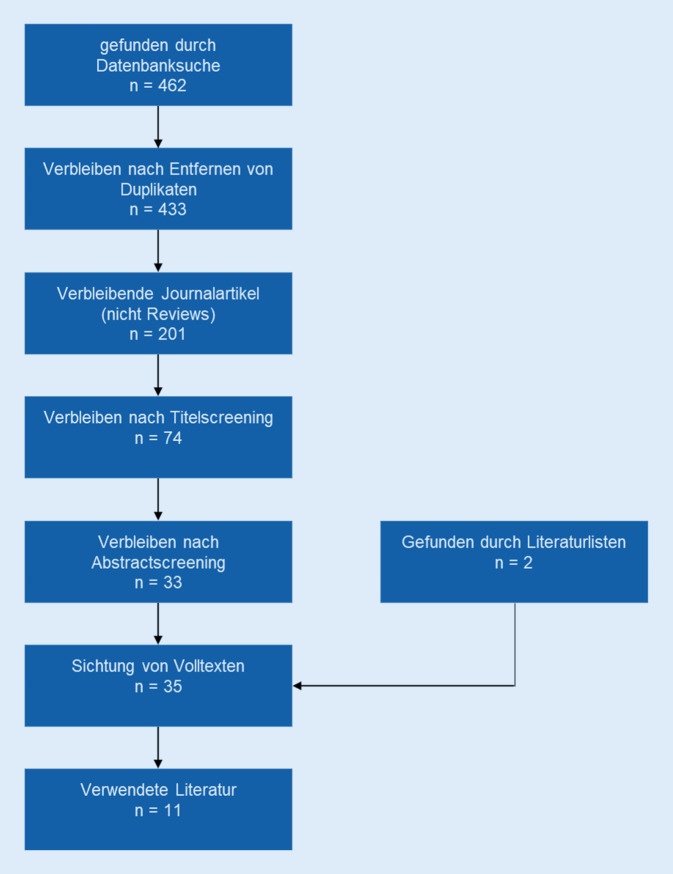

Die Literaturrecherche ergab 433 unikale Treffer. Zunächst wurden, den formalen Ein- und Ausschlusskriterien folgend, alle Reviews, Buchkapitel und Konferenzbeiträge ausgeschlossen (232 Beiträge). Bei den verbleibenden 201 Artikeln wurden die Titel und anschließend die Abstracts thematisch mit den Suchkriterien abgeglichen und dabei beispielsweise rein technische Artikel zur Funktionsweise von Sensoren oder Robotern ausgeschlossen. So konnten 11 Artikel final in die Auswertung einbezogen wurden (Abb. 1). Eine Zusammenfassung der eingeschlossenen Artikel gibt Tab. 1. Die Artikel wurden thematisch nach (1) Potenzialen mit den Unterpunkten Einsamkeit, Kommunikation sowie Stress und Stimmung und (2) Herausforderungen mit den Unterpunkten technische und kontextuelle Faktoren sowie ethische Grundsätze, inkl. der Reduktion sozialer Beziehungen, geordnet. Den überwiegenden Fokus stellten pflegerische Settings (z. B. bei demenziell Erkrankten) dar; nur vereinzelt wurde der Einsatz von sozialen Robotern bei gesundheitlich nicht- bzw. wenig funktionseingeschränkten älteren Menschen beforscht.

**Table 1:** 

Autor/-innen (Jahr)	Artikelart und Methode	*n*	Thema/Fragestellung	Variablen	Roboter	Wesentliche Ergebnisse
Baisch et al. (2018) [1]	Empirisch: 2 Mixed-methods-Studien	30	Studie 1: Erfahrungsanalyse über den Einsatz von Paro bei Pflegekräften im stationären Setting	Einsatzkontext und -häufigkeit	Paro & Pleo	Studie 1: wöchentlicher Einsatz von Paro als therapeutisches Mittel und Beschäftigungsangebot. Gepflegte verkennen Paro als lebendig, was nicht aufgeklärt wird
		45	Studie 2: Beschäftigung mit Pleo für 15 Tage in der eigenen Häuslichkeit bei Personen mit geringen Einschränkungen	Akzeptanz		Studie 2: sowohl hohe Akzeptanz von Pleo als auch Angst und Vorbehalte bei Personen mit psychischen Auffälligkeiten. Gewöhnung und Erwartungen fördern Akzeptanz
				Reaktionen auf Roboter		
Bemelmans et al. (2015) [2]	Empirisch: quasiexperimentelle Zeitreihe	71	Einsatz von Paro bei Demenzerkrankten im Pflegeheim zur Therapie und zur Pflegeunterstützung	„Psychological functioning well-being“	Paro	Der therapeutische Einsatz von Paro erhöht das Functioning und die Lebenszufriedenheit von Demenzerkrankten – der Einsatz zur Pflegeunterstützung hat keinen Effekt auf die Outcomes. Paro sollte als Werkzeug von Pflegenden benutzt werden und kann die Pflegekräfte nicht ersetzen
Chen et al. (2019) [4]	Empirisch: Interviews und Survey	I: 8	Identifikation von Lebenssituationen, in denen Gesellschaft erwünscht ist, und Abgleich mit technischer Umsetzbarkeit	Identifikation von Lebenssituationen, Bewertung nach Wunsch nach Gesellschaft	Soziale Roboter generell	Der stärkste Wunsch nach Gesellschaft durch soziale Roboter besteht beim Essen, bei der Hausarbeit und der Gesundheitsförderung. Dies sind jedoch nicht die Bereiche, die technisch am einfachsten lösbar sind, sodass es zu einem Ungleichgewicht zwischen Nachfrage und Angebot kommen kann
		S: 234				
De Graaf et al. (2015) [6]	Empirisch: Interviews	6	Roboter zur Gesundheitsförderung wurden 10 Tage lang in der Häuslichkeit der Proband*innen installiert, um Einflussfaktoren auf die Akzeptanz zu ermitteln	Nützlichkeit	Harvey	Nicht die instrumentellen, funktionalen Elemente, sondern besonders spielerische, beziehungsbildende Aspekte sind relevant für die Akzeptanz der Roboter
				Bedienbarkeit		
				Hedonismus		
Khosla et al. (2019) [11]	Empirisch: Beobachtungen, Interviews	5	Roboter wurden 3 Monate in der Häuslichkeit von Demenzkranken installiert	Beschäftigung	Betty	Personalisierte Services werden gegenüber standardisierten bevorzugt; wenn der soziale Kontext im Design berücksichtigt wird, ist der Beziehungsaufbau möglich, sodass die Roboter Interaktionen initiieren und erfolgreich abschließen können
				Einstellungen		
				Nützlichkeit		
				Sorge		
Khosla et al. (2017) [12]	Empirisch	115	Roboter wurden in 4 Pflegeheimen bei Demenzerkrankten über 4 Jahre hinweg eingesetzt	Beschäftigung, Einstellungen, Bedenken	Matilda	Die Demenzerkrankten beschäftigten sich auch nach einem Jahr stärker mit Matilda (visuell, emotional und spielerisch). Zudem wurde Matilda positiv in Bezug auf Einstellungen evaluiert, und nur 2 % der Teilnehmer/-innen schilderten Bedenken hinsichtlich der Anwesenheit von Matilda
Körtner (2016) [13]	Theoretisch	N/A	Ausgehend von ethischen Prinzipien werden wesentliche Risiken beim Einsatz von sozialen Robotern bei Älteren diskutiert	Nichtschaden, Wohltätigkeit	Sozial-assistive Roboter generell	Ethische Prinzipien – Nichtschaden, Wohltätigkeit, Autonomie und Fairness – sollten fundamentaler Teil sozialer Roboter und ihrer Umsetzung sein. Wesentliche Risiken bestehen bei den Themen Würde, Täuschung und Privatsphäre, Isolation, Verletzlichkeit und Sicherheit
				Autonomie		
				Fairness		
				Würde		
				Täuschung		
				Privatsphäre		
				Isolation		
				Verletzlichkeit		
				Sicherheit		
Lorenz et al. (2016) [14]	Theoretisch	N/A	Synchronität und Reziprozität werden als zentrale Mechanismen im Beziehungsaufbau identifiziert und auf die Mensch-Roboter-Interaktion übertragen	Synchronität	Companion (Begleit‑)Roboter	Synchronität und Reziprozität können die Entwicklung sozialer Roboter vorantreiben und bessere Ergebnisse bei Therapien und Rehabilitationen, die soziale Roboter einsetzen, erzielen
				Reziprozität		
Robinson et al. (2013) [18]	Empirisch: „randomized controlled trial“	40	Vergleichende Bewertung von Robotern vs. „resident dog“ in Pflegeheimen über 12 Wochen	Einsamkeit	Paro	Paro hat Einsamkeit bei Teilnehmer/-innen reduziert und wird als positiv betrachtet, auch im Gegensatz zum Resident dog. Depression und Lebensqualität waren unverändert
				Depression		
				Lebensqualität		
Šabanović und Chang (2016) [19]	Theoretisch	N/A	Robotische Sozialität wird anhand eines Roboters in einem Versuchsraum sowie in einem Pflegeheim untersucht	Sozialer Kontext	Paro	Der soziale Kontext (durch weitere Menschen in der Interaktion) ist wichtig für die erfolgreiche Gestaltung und Implementierung von Paro
				Sozialität		
Sharkey und Sharkey (2012) [20]	Theoretisch	N/A	Identifikation von ethischen Themenfeldern beim Einsatz von sozialen Robotern bei Älteren	Verlust menschlichen Kontakts	Assistive Roboter, Companion (Begleit‑)Roboter	Vorteile in der Pflege durch soziale Roboter werden mit den 6 ethischen Themenfeldern abgewogen. Bei reflektierter Umsetzung können soziale Roboter das Leben älterer Menschen bereichern
				Objektifizierung		
				Privatsphäre		
				Freiheit		
				Täuschung und Infantilisierung Menschliche Kontrolle der Roboter		

### Potenziale

Die Potenziale sozialer Roboter wurden bisher theoretisch erörtert und in einigen Studien mit dem Fokus auf den Pflegekontext empirisch gezeigt. Im Hinblick auf beziehungsrelevante Aspekte des höheren Alters gliedern sie sich in die Themen Einsamkeit, Kommunikation sowie Stress und Stimmung.

#### Einsamkeit.

Ein Versprechen sozialer Roboter ist es, Einsamkeit als subjektiv wahrgenommenes Alleinsein zu reduzieren [20]. In Pflegeheimen konnte gezeigt werden, dass Paro Einsamkeit über die Zeit und im Vergleich zu einer Kontrollgruppe ohne zusätzliche Beschäftigung reduzierte – allerdings mit vergleichsweise wenigen Teilnehmer/-innen (*n* = 40), wodurch die Aussagekraft dieser Befunde eingeschränkt wird [18]. Chen et al. [4] zeigten darüber hinaus, dass soziale Roboter eine Bereicherung in den Situationen darstellten, in denen Gesellschaft erwünscht ist (z. B. beim Essen).

#### Kommunikation.

Soziale Roboter können einerseits die Kommunikation *mit ihnen* fördern, indem sie Gespräche initiieren. Andererseits fördern sie, im therapeutischen Gruppensetting eingesetzt, die zwischenmenschliche Kommunikation *über sie*, was wiederum die soziale Integration stärken kann [20]. In ihrer 4‑jährigen Studie mit dem sozialen Roboter Matilda konnten Khosla et al. [12] lediglich in wenigen Jahresvergleichen geringe, jedoch signifikante Steigerungen der verbalen, nonverbalen und behavioralen Beschäftigung (Engagement) bei 115 Demenzpatienten/‑patientinnen feststellen. In der Studie von Robinson et al. [18] zeigte sich ebenfalls mehr Kommunikation mit und über den Roboter mit anderen Menschen.

#### Stress und Stimmung.

Durch verstärkte soziale Interaktion und die Reduktion von Einsamkeit kann ebenfalls die Ausschüttung von Stresshormonen reduziert werden, was zur Stressreduktion in sozialen Beziehungen und zur Stimmungssteigerung führen kann, die damit weitere Versprechen sozialer Roboter in theoretischen Überlegungen sind [20]. Nach der Beschäftigung mit dem Roboter Paro wurden empirisch bei 71 Demenzpatient/-patientinnen bessere Werte auf einer Stimmungsskala (Mood Scale) und bei der Erfüllung individueller Therapieinterventionen gefunden [2].

### Herausforderungen

Herausforderungen beim Einsatz sozialer Roboter ergeben sich aus technischen und kontextuellen Faktoren, zu denen Reziprozität und die soziale Einbettung zählen, sowie aus ethischen Überlegungen, die eine Orientierung an Prinzipien und Leitlinien ebenso einschließen wie eine kritische Reflexion über eine drohende (weitere) Reduktion sozialer Beziehungen. In der bestehenden Literatur gilt spezielles Augenmerk der Vulnerabilität der Zielgruppe, besonders in pflegerischen und therapeutischen Settings, sowie der Frage, ob soziale Roboter überhaupt langfristige (positive) Wirkungen hervorrufen können.

#### Technische und Kontextfaktoren.

Aus theoretischer Sicht werden als Voraussetzungen für den erfolgreichen Einsatz von sozialen Robotern die Synchronität von Bewegungen, d. h. gleichzeitiges Ausführen derselben oder komplementärer Handlungen zweier Akteure, die Reziprozität, also ein als gleichberechtigt wahrgenommenes Geben und Nehmen [14] und der soziale Kontext der Interaktion gesehen [19]. Hierbei wird Wert daraufgelegt, dass die Interaktion nicht rein technisch im sozial luftleeren Raum stattfindet, wie es oftmals einer rein ingenieurwissenschaftlichen Herangehensweise entspricht, sondern durch weitere Personen sozial geprägt ist. Spielerische Elemente und die Nachahmung menschlicher Charakteristika (Anthropomorphismus) seien, theoretischen Überlegungen folgend, notwendig, damit ein Beziehungsaufbau in der Mensch-Roboter-Interaktion entstehen könne [14, 19]. Die Empirie untermalt diese theoretischen Überlegungen und zeigt, dass hedonistische Elemente sozialer Interaktion wie Spaß oder Anthropomorphismus ins Zentrum gestellt werden sollten [6]. Ebenfalls empirisch zeigt sich, dass für den Erfolg eines sozialen Roboters bei der Aktivierung von Ressourcen weniger die technischen Komponenten der Maschine wichtig seien, sondern vielmehr die soziale und kontextgebundene Einbettung durch beispielsweise Pflegende [1, 11].

#### Ethische Grundsätze.

Die ethische Bewertung sozialer Roboter folgt ausschließlich theoretischen Überlegungen – empirische Studien, die untersuchten, inwieweit die vorgeschlagenen ethischen Konzepte berücksichtigt wurden, konnten in diesem Rapid review nicht gefunden werden. Bei der ethischen Bewertung der Anwendung sozialer Roboter schlagen Autor/-innen vor, die Prinzipien Nichtschaden, Wohltätigkeit – die Bestrebung, mögliche Nachteile bei Nutzer/-innen abzuwenden, Autonomie und Fairness anzulegen [13] und die Privatsphäre der Menschen, ihre Würde sowie ihr Recht, nicht getäuscht oder bevormundet zu werden, zu berücksichtigen [13, 20]. Hieraus ergebe sich, so die Autor/-innen, ein Korridor für den Einsatz sozialer Roboter, beispielsweise mit den Zielen, die Einsamkeit zu reduzieren und die Ressourcen der Benutzer/-innen zu aktivieren [13, 20]. Sharkey und Sharkey [20] sehen die Übernahme sozialer Aufgaben von Technik indes als Rückzug menschlichen Interagierens, der im Konflikt mit den oben beschriebenen Prinzipien stehen kann. Sie raten entsprechend, soziale Roboter als Ergänzung zur menschlichen Interaktion einzusetzen, jedoch nicht als Ersatz, um Versorgungsengpässe zu minimieren. Letztlich wird das Thema Verantwortung als offene Frage aufgeworfen, besonders wenn Fehler in der Nutzung der Roboter auftreten (wie z. B. technische/funktionale Insuffizienz oder fehlerhafte Benutzung durch die Anwender/-innen) [20].

## Schlussfolgerung

Dieses Rapid review zeigte positive Auswirkungen des Einsatzes von sozialen Robotern auf Bereiche wie Einsamkeit, Kommunikation, Stress und Stimmung, die in Verbindung mit sozialen Beziehungen stehen. Allerdings gibt es noch keine ausreichende empirische Evidenz darüber, wie genau sich soziale Roboter v. a. langfristig auf die Ressourcen und die Netzwerkinteraktionen älterer Menschen auswirken. Potenziale zeigten sich v. a. in verringerter Einsamkeit, mehr Kommunikation und Stressreduktion. Diese sind jedoch größtenteils im institutionellen pflegerischen Setting mit kurzen Beobachtungsphasen entstanden. Empirische Studien zum Einsatz von sozialen Robotern in der eigenen Häuslichkeit sind unterrepräsentiert und haben zu geringe Fallzahlen, um aussagekräftige Befunde zu erzeugen. Aus dieser Forschungslücke wird ein Bedarf abgeleitet, die bestehende Literatur um Studien in der eigenen Häuslichkeit zu ergänzen.

Daneben identifizierte das Rapid review Herausforderungen in diesem Feld. Hier stehen die Betrachtungen von Kontextfaktoren und ethische Überlegungen zum Einsatz von Robotern im Vordergrund. Der Einsatz sozialer Roboter, der nicht an den individuellen Bedürfnissen der Menschen orientiert ist, kann sich negativ auf die Ressourcen sowie die Qualität und Quantität sozialer Beziehungen älterer Menschen auswirken. Daher sollte der Einsatz besonders in der Erprobungsphase dieser neuen Technologie stets kritisch begleitet werden und, wo irgend möglich, ältere und alte Menschen selbst miteinbeziehen. Aufgrund der geringen Anzahl empirischer Befunde wird hier ein großer Forschungsbedarf gesehen, um die Grenzen sozialer Roboter besser beschreiben zu können.

## Fazit für die Praxis

- Im konkreten Anwendungsfall sollten Pflegende und Angehörige Chancen und Risiken sorgfältig und kontextbezogen abwägen und darauf achten, dass soziale Roboter sich nicht negativ auf beziehungsrelevante Ressourcen sowie soziale Beziehungen Älterer auswirken.
- Designer und Hersteller sozialer Roboter sollten technische Voraussetzungen und ethische Aspekte in der Konzeption und Produktion berücksichtigen, um die Potenziale ausschöpfen zu können.
- Die Forschungspraxis sollte den bisher vernachlässigten Anwendungsfall sozialer Roboter bei wenig funktionseingeschränkten Menschen in der eigenen Häuslichkeit betrachten.
- Die politischen und institutionellen Entscheider/-innen tragen Verantwortung, die mit der Technisierung und Digitalisierung von Pflege und Versorgung verbundenen Werthaltungen, die vermittelt werden, zu reflektieren und ggf. regulierend einzugreifen.

## Supplementary Information




